# Virtual Reality Versus Simulation in the Management of Trauma Based Scenarios—A Systematic Review

**DOI:** 10.1002/hsr2.70216

**Published:** 2024-12-04

**Authors:** Manal Ahmad, Mi‐Tra Tran, Basma Ahmad, Konstantinos Kavallieros, Joseph Shalhoub, Alun Huw Davies

**Affiliations:** ^1^ Section of Vascular Surgery, Department of Surgery and Cancer Imperial College London London UK; ^2^ Imperial Vascular Unit, Imperial College NHS Trust London UK; ^3^ School of Medicine Imperial College London London UK; ^4^ University Hospital Plymouth NHS Trust Plymouth UK

**Keywords:** medical education, simulation, trauma, virtual reality

## Abstract

**Introduction:**

Simulation allows trainees to practice skills safely. It is the current gold standard method of teaching. More recently, novel methods such as virtual reality and augmented reality are being explored as possible alternative methods.

**Aims:**

To evaluate the current evidence pertaining to simulation and virtual reality as methods of teaching in teaching trauma management.

**Methods:**

Medline and Embase (via Ovid interface) were used to search for articles up to April 2023. A combination of the following MeSH terms were employed in the primary search string ‐ “virtual reality,” “simulation,” “surgery,” “trauma,” and “medical education.”

**Results:**

3815 studies were initially identified. After de‐duplication, 2648 articles were screened using Covidence. Forty articles underwent full text review. Thirteen studies were included in the final review with a pooled total of 489 participants. Significant heterogeneity exists in the range of participants, scenarios and parameters assessed. The overall self‐reported perception of VR as a teaching modality is positive and is well accepted however objective assessment and validation is needed.

**Conclusion:**

VR can be useful for training and evaluation of trauma‐based scenarios. It is a useful adjunct but is unlikely to replace simulation at present. More robust and replicable studies with larger sample sizes are needed to evaluate the long‐term integration of virtual reality and augmented reality into the medical and surgical teaching curriculum.

## 1 Introduction

Trauma from unintentional and intentional injuries leads to over five million deaths annually [1]. It is responsible for life altering disabilities which can have a profound impact on the lives of those involved. The assessment and management of trauma in pre‐hospital and hospital environments is based on the Advanced Trauma Life Support (ATLS) principles. These have continued to evolve since its first iteration in 1978 [1]. Medical training is primarily based on the apprenticeship/mentorship model and grounded in experiential learning on the road to competency [2, 3, 4]. Trauma, given its high stakes, increases the overall risk to patient safety in matters of learning and training. The acute nature of trauma demands accurate assessment, management and decision making in a time critical manner. It also requires regulation of essential nontechnical skills that is, communication, leadership, active listening, teamwork and stress management (within oneself and the team). These technical and nontechnical skills often need to work synchronously and in synergy.

Simulation training is a commonly employed teaching method in the medical field. It offers a safe arena for professionals to practise and hone their skills in a controlled environment and provides the opportunity for replication of real‐world scenarios in an interactive manner. It is utilized in enhancing the training experience, developing problem solving and decision making skills whilst also improving interpersonal skills. Simulation also allows for robust assessment in areas where stakes are often high [5]. It is currently regarded as the “gold standard” method of teaching and assessing the management of trauma in courses including ATLS, advanced cardiac life support and objective structured clinical examinations (OSCE's).

The term virtual reality (VR) was first coined by Jaron Lanier in 1898 however, the concept of a display capable of reflecting the world we live in was proposed by Sutherland almost six decades ago [6]. The first VR headset–Oculus Rift ‐ arrived into the public consciousness in 2012 and has since been applied to a multitude of disciplines including medicine. Whilst the costs of the initial hardware made it a novelty, current costs and the general availability of hardware such as the Oculus Headsets, HTC Vive, PlayStation VR and Apple Vision Pro, makes it accessible and could be utilized as a method for applied teaching in the field of trauma. VR can also be delivered via virtual worlds through a personal computer‐based interface. It allows immersion in a virtual setting using 360 degree video cameras, head mounted displays, synthetic graphics and audio [7].

## 2 Aims

The aim of our review was to identify and evaluate the existing literature pertaining to the effectiveness and usefulness of virtual reality and simulation as a modality for teaching the management of acute trauma‐based scenarios.

## 3 Methods

Our review was performed in accordance with the preferred reporting for systematic reviews and meta‐analyses statement [8]. The literature was searched using Embase and Medline (via Ovid interface) databases. A combination of the medical subject headings terms “virtual reality,” “simulation,” “surgery,” “trauma” and “medical education” were utilized in the primary search strategy.

The inclusion criteria consisted of randomized control trials, cohort studies, cross‐sectional studies and observational studies including trauma in the context of the ATLS curriculum. The participants of interest in the studies included medical students, trainee doctors/residents and healthcare specialists. Studies were limited to the English language. No time limit was placed for the search. All studies using virtual reality and/or simulation for the management of trauma‐based scenarios across all medical, surgical and paramedical specialities were included.

Our exclusion criteria consisted of case series, abstracts, expert opinions, orthopedic trauma‐based studies pertaining to operative interventions i.e hemiarthroplasty, internal/external fixation and surgical management in the form of endoscopic, laparoscopic or neurosurgical procedures.

The abstract and title screening and full text review was completed using the Covidence software by two reviewers (M.A. and M.T.). Data extraction was completed by three reviewers (M.T., B.A., K.K.) using an excel spreadsheet. We assessed the studies for risk of bias using the Newcastle Ottawa Scale [9].

## 4 Results

**Figure 1:**
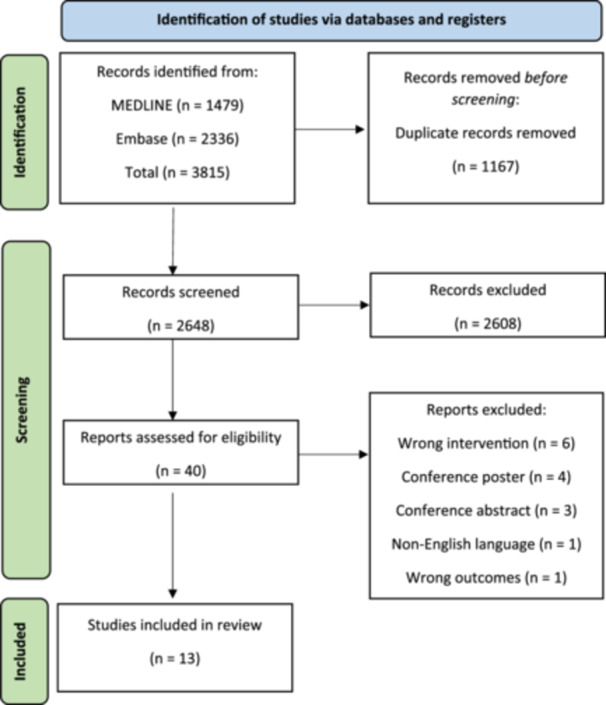
PRISMA flow diagram.

3815 studies were initially identified. After de‐duplication, 2648 articles were screened using Covidence. Forty articles were included in the full text review. Thirteen studies met the criteria for our review and underwent data extraction (Figure 1).

A pooled total of 489 participants were included from the studies in the review, the majority of which were prospective cohort studies followed by randomized control trials. The studies spanned from 2008 to 2023 and are summarized in Tables 1, 2, 3. The risk of bias was high for the studies included (Table A1). The VR programs were delivered either via computer based virtual worlds or via immersive head mounted displays.

**Table 1:** Study characteristics.

Author	Year	Country	Study design	Number of participants	Male:Female ratio	Participants
**Arif et al.**	2023	USA	Prospective RCT	40	27 M:13 F	Emergency Medicine Technician students
**Bowyer et al.**	2008	Iraq	Cross‐sectional	65	NOS	Combat medics
**Brenner et al.**	2014	USA	Prospective Cohort	13	NOS	Novice REBOA interventionalists (board certified in general surgery, surgical critical care, cardiothoracic, critical care, emergency medicine without previous endovascular teaching)
**Cohen et al.**	2013	UK	Prospective cohort feasibility study	23	NOS	12 Ambulance HART 6 Trauma team leaders (ATLS‐certified) 5 Accident and Emergency consultants
**Colonna et al.**	2022	USA	Cohort study	31	17 M:14 F	64th year medical students 18 general surgery residents (year 1–year 6) two flight nurses four acute care surgeons one surgical oncologist
**Couperus et al.**	2020	USA	Prospective Cohort	10	NOS	Working group comprised of active duty/former military emergency medicine physicians
**Courteille et al.**	2018	Sweden	RCT	170	93 M:77 F	85 4th year medical students + 85 orthopedic residents
**Hainsworth et al.**	2022	UK	RCT	14	NOS	Foundation doctors
**Harrington et al.**	2018	Ireland	Cohort study	26	21 M:5 F	18 ATLS candidates + 11 ATLS instructors
**Kiyozumi et al.**	2022	Japan	Observational study	14	NOS	Five medical students + nine paramedical students
**Proctor et al.**	2014	USA	RCT	32	NOS	Army (combat medical doctors)
**Pucher et al.**	2014	UK	Cohort study	21	NOS	Seven novice (junior residents), eight novices (residents), six experts (consultants/attendings)
**Youngblood et al.**	2008	USA	RCT	30	15 M:15 F	13 postgraduate doctors + 17 medical students

Abbreviations: HART, ambulance hazardous area response team; NOS, not otherwise specified; RCT, randomized control trial.

**Table 2:** Summary of studies.

Author	VR	Scenario	Comparator	Parameters assessed/evaluated
**Arif et al.**	Oculus Quest 2	Tourniquet hemorrhage control	Simulated tourniquet hemorrhage control	1. New York State Emergency Medical Technicians certification of skills rubric 2. Pre/post intervention survey
**Bowyer et al.**	Computer based software + crystal eyes shutter glasses + haptic interface devices (Two Phantom Omni)	Cricothyroidotomy	None	Feasibility study 1. Self‐reported questionnaire (realism, perceived level of comfort)
**Brenner et al.**	Computer‐based software with haptics (Vascular Intervention System Training Simulator‐C)	Resuscitative Endovascular Balloon Occlusion of the Aorta (REBOA)	None	1. OSCE 2. Time to complete procedure 3. Subjective global rating (Likert scale 1–5) based on task performance
**Cohen et al.**	Computer‐based software (Second Life simulator)	X1 Pre‐hospital and X2 In‐hospital scenarios ‐ Blast injury amputation + occult cervical spine fracture	None	Feasibility study 1. Self‐reported questionnaire
**Colonna et al.**	Oculus Quest 2	Single blunt polytrauma resuscitation	None	1. Identification and number of critical decision points (10 steps ‐ intubation, cricothyroidotomy, chest tube, intravenous access, focused abdominal sonography for trauma examination, pelvic binder, activation of massive transfusion protocol, administration of hypertonic saline, hyperventilation and decision take the patient to the operating theater) 2. Time taken to make correct decision 3. Self‐reported questionnaire – acceptance of VR and satisfaction using the fast form of the technology acceptance model
**Couperus et al.**	Immersive VR (Microsoft Mixed Reality – Exonicus Inc, Anatomy Next Inc, Kitware Inc)	Hemorrhage Tension Pneumothorax Airway Obstruction	None	1. OSCE grading based on Joint Trauma Committee Clinical Practice + ATLS guidelines 2. Working group feedback
**Courteille et al.**	Virtual patient	Cervical spine trauma	Didactic teaching (video of lecture)	1. Multiple choice questions 2. Self‐reported questionnaires
**Hainsworth et al.**	VR fully immersive interactive video teaching	Two trauma calls: road traffic accident with lower abdominal pain and fall with deformed and shortened leg	Didactic teaching	1. Time taken for essential tasks (extrapolated from ATLS guidelines) 2. Self‐assessment questionnaire
**Harrington et al.**	Samsung gear VR head mounted display ‐ Oculus, (Unity 3D software)	Blunt thoracic trauma	Instructor led teaching session	1. Simulator variables (patient deaths, decisions made, diagnoses made) 2. Multiple choice questions
**Kiyozumi et al.**	IDEALENS K4 hardware (CREEK & RIVER software)	Prehospital trauma scenario	None	1. Japan Prehospital Trauma Evaluation and Care (JPTEC) assessment by instructors 2. Number of correct decisions made 3. Time taken to complete scenario
**Proctor et al.**	Phantom Omin haptic interface devices (HapMed and CricSim software)	Cricothyroidotomy	Mannequin	1. Davis Technology Acceptance Model (TAM) framework 2. Questionnaires to evaluate effectiveness, usability
**Pucher et al.**	Virtual World with live communication (Unity 4 games engine software)	Mass casualty incident three scenarios	None	1. Patient transfer/critical decisions 2. Time to complete assessment and make decision 3. Trauma‐NOnTECHnical Skills (T‐NOTECHS) score
**Youngblood et al.**	Computer‐based software with haptics and live communication (Atmosphere (Adobe Systems), Poser (Curious Labs Inc) software)	Trauma (pretest case followed by four training cases and a posttest case)	None	1. Emergency Medicine Crises Resource Management (EMCRM) + ATLS based scores 2. Leadership scores (three raters)

**Table 3:** Summary of findings.

Author	Findings	Statistical analysis	*p*‐values
**Arif et al.**	1. No significant difference in initial correct tourniquet skill performance between control and VR 2. Final correct tourniquet skills performance 3. No significant difference on average number of errors made during initial assessment 4. Final skills assessment 5. VR 12.27 times more likely to fail due to improper tightening during final assessment compared to initial 6. VR 8.39 times more likely to apply gloves in final assessment compared to initial.	Binomial logistic regression (successful tourniquet placement, error in tourniquet application), Linear regressions (number of total errors, time to apply a tourniquet), ordinal logistic regressions (survey data), One‐way ANOVA (difference in total number of error during final assessment between the 3 classes)	(1) 89% versus 76%, *p* = 0.42 (2) 63% versus 57%, *p* = 0.57 (3) 0.11 versus 0.24, *p* = 0.30 (4) 1 versus 0.85, *p *= 0.81 (5), *p* = 0.04 (6), *p* = 0.01
**Brenner et al.**	1. Significant decrease in procedural task time between task 1 and 6. 2. Significant improvement in knowledge as assessed pre and posttest 3. Significant shorter task 1 procedural time for those who play video games, but not at task 6 4. No correlation between test scores and procedural time and potential predictors (e.g. experience) 5. 85% strongly agreed they were prepared to perform REBOA on their next call.	n/a	(1) 119.7 s–176.0 s versus 46.6 s, *p* < 0.0001 (2), 0.92–2.92 versus 1.66, *p* = 0.0013 (3), *p* = 0.03
**Cohen et al.**	87% felt VR orientation provided was adequate; 91% could use the interface easily; “agreed” or “strongly agreed” that scenarios were realistic visually (87%) and clinically (96%). In‐hospital scenarios would have benefitted more from environmental stressors and distractions. 95% would use a similar sim again; and recommend to colleagues.	n/a	n/a
**Colonna et al.**	1. Less experienced learners have higher satisfaction. 2. Most decisions made correctly by seniors and acute care surgeons. 3. Most found simulation comfortable (3.67/4 points), clinical reasoning and clinical learning scores approached 4 out of 5. 4. High scores for efficiency and effectiveness, moderative‐high for ease of using and learning tech.	Fischer's exact test (categorical comparisons)	(1) *p* = 0.039
**Couperus et al.**	1. Working group unanimously indicated high level of realism and potential training usefulness. 2. Worked without internet ‐ highlights capability of autonomous running.	n/a	n/a
**Courteille et al.**	1. No significant difference in knowledge acquisition implying similar short‐term retention on both groups 2. Small significant decline in knowledge retention over time in both groups 3. (3) no sig‐diff in long term retention test; High median values for engagement, stimulation, general opinion and learning expectations in both groups.	Repeated‐measures analysis of variance for knowledge scores	(2) *p *= 0
**Hainsworth et al.**	1. VR group average 1min 58s faster at completing each essential task. 2. Decreased anxiety levels when attending trauma call ‐ standard teaching 6.3/10, VR 4.4/10 3. (3) VR group felt more prepared to attend trauma call 8/10 versus 2.9/10.	n/a	n/a
**Harrington et al.**	1. Instructor group significantly less deaths 2. Lower proportions of incorrect decisions than candidate group 3. No sig diff in time taken or no. correct diagnoses 4. MCQ performance did not correlate with simulator performance in overall candidate group. 5. (5) enjoyable method of learning (median rating 6.0), the learning platform of choice (median 5.0) and a cost‐effective training tool (median 5.0)	Student's t‐test for continuous data, Fisher's exact test for categorical data, Spearman's Rank correlation for MCQ assessment	(1) *p* = 0.049 (2) *p* = 0.045 (3) *p* = 0.92, *p* = 0.882
**Kiyozumi et al.**	1. They “cleared” the scenario 13 times (median) in 15 min. The median number of times the learners cleared the scenario was three in the first 5 min, 5 in the second 5 min, and five in the third 5 min. 2. The results of multiple comparisons in the Bonferroni correction were: *p* = 0.0125 between the first 5 min and the second 5 min, 0.0915 between the second 5 min and the third 5 min, and *p* = 0.0045 between the first 5 min and the third 5 min. The increase in clearance rate with time up to 10 min was statistically significant. 3. All participants passed the practical skills evaluation.	The differences between groups were tested using the Friedman test and Bonferroni correction	(1) *p* < 0.001
**Proctor et al.**	1. User recommendation: 23 out of 26 (88.46%) responded positively for CricSim; 24 (92.3%) answered positively for HapMed 2. Visual interaction within CricSim is more clear andunderstandable than HapMed	Wilcoxon paired sign rank test on matched pairs	(2) *p* = 0.00338
**Pucher et al.**	1. Greater number of risk or critical events identified in the junior group, 11 versus 3 2. Mean (SD) time from patient arrival to disposition was significantly higher in the junior group, 560 (299) seconds versus 339 (321) seconds 3. Trauma‐NOnTECHnical Skills (T‐NOTECHS) score for nontechnical ability were lower in the junior cohort, 14.0 (2.0) versus 21.5 (3.7) 4. Mean technical scores assessing the completion of tasks relating to each subject's role responsibility were also lower in the junior group, 2.29 (0.34) versus 3.96 (0.69) 5. All universally agreeing that the simulation was effective and realistic	Chi‐squared Mann‐Whitney U	1. *p* = 0.006 2. *p* = 0.026 3. *p* = 0.003 4. *p* = 0.001
**Youngblood et al.**	1. Subjects who used either the Virtual ED or the simulation showed significant improvement in performance between pretest and posttest cases (*p* < 0.05). 2. No significant difference in subjects' performance between the two types of simulation 3. Data on usability and attitudes toward both simulation methods as learning tools were equally positive.	Wilcoxon signed rank test, a nonparametric test for statistical significance of scores between two groups.	Significant improvement in performance between pretest and posttest cases (*p* < 0.05)

There was significant heterogeneity of the participants across the studies and included undergraduate medical students, postgraduate medical doctors from a range of backgrounds–junior doctors/residents (non‐specialized), consultants, army medics, surgeons, emergency department physicians, emergency medicine technicians and paramedics [10, 11, 12, 13, 14, 15, 16, 17, 18, 19, 20, 21].

The focus of the included trauma scenarios varied significantly with hemorrhage control, blast injuries, blunt trauma, cervical spine fractures, airway issues and mass casualty incidents. 4 of the 12 included studies had a comparator which included simulated teaching stations, didactic teaching and mannequin‐based simulation [10, 15, 16, 17, 19]. 6 studies had teaching sessions or induction before using VR teaching modalities [10, 11, 16, 20, 21]. There was significant heterogeneity in the evaluation methods and parameters assessed however, these can broadly be categorized into objective decision‐based rubric scoring, multiple choice questions, time‐based assessments, self‐reported questionnaires and surveys.

## 5 Discussion

The general reported feedback pertaining to VR is very positive. Participant's impression of interaction, engagement and self‐perceived improvement scores increased in the vast majority of post VR surveys [10, 11, 13, 20]. Most participants readily recommended VR as a training modality for other residents and expressed a willingness to use virtual environments for future training, agreeing that the visual portrayal and scenarios were realistic and enjoyable [11, 12, 14, 19, 20, 22]. Feedback from earlier iterations have suggested improving the graphics to produce a realistic immersive experience however, the studies included in the review often fail to have further follow‐up or use of the VR programs which renders it difficult to assess for any evolution beyond the pilot projects [17]. In general, participants seem to accept VR as an adjunct to aid revision and this is reflected in the likert based scores and questionnaires. Colonna and Proctor used alternative methods to assess user acceptibility including the fast form of the technology assessment model and the technology acceptance model (TAM) framework [13, 19, 23]. TAM is based on two measures–perceived usefulness and ease of use. It is important to note that this is based on the user's personal perceptions and can vary across demographics and time as technology evolves and more complex versions become integrated into day‐to‐day life as a norm. The fast form TAM (FF_TAM) is a validated semantic differential scale which assesses the same constructs as the original TAM [24]. Whilst Likert scales are grounded in the extent of agreement with statements, semantic differential scales measure connotative meaning and provide greater freedom by allowing the use of different labels and ratings [24]. It is however worth noting that the TAM and FF_TAM were created at a time preceding the era of social media, the influence of which cannot be ignored. The updated iterations of TAM and FF_TAM, the Unified Theory of Acceptance and Use of Technology 2 (UTAUT2) factors in other influences including performance expectancy, effort, social, environment, hedonic motivation, price and habit into the overall perception and use of any technology [25, 26, 27, 28]. VR can offer realistic, simulated scenario algorithms however, given that all studies created their own in‐house software, it would be imperative to attempt trials using the software in other groups and settings for external validation. Another aspect of interest to consider is the evaluation of VR through a consumer‐based lens as an integrative adjunct in the realm of medical education accounting for these external influences. In the era of dynamic experimental and integrative artificial intelligence, the perception and adaptation of VR in medical education could allow personalization of training needs from a multidisciplinary perspective in a manner which would otherwise be cost and resource heavy.

General accessibility to remote VR teaching offers the benefit of allowing participants to revisit the information over time which is not the case in didactic/in‐person simulation training and can lead to an increased perception in preparedness and reduction in anxiety levels [16]. It can also improve self‐reported levels of confidence, independence and leadership scores [18, 21]. VR does offer benefits in training exercises which can otherwise be costly. The feasibility of an evidence based virtual environment using a web‐based application and involving multidisciplinary team members in preparing for a major incident response is explored as an alternative to running live exercises by Cohen et al [12]. This was found to be user friendly, reflective of real‐world practice and was well received by participants. Web‐based applications including Second Life and Open Simulator were low cost and easily accessible without geographical constraints. Interestingly, the VR group were found to have a statistically significant improvement in knowledge along with a reduction in the procedural task time regardless of other confounding factors in the use of REBOA [11]. VR also allows accessibility to teaching resources which can aid revision and can help individuals feel prepared through repetition and self‐reflection to manage trauma based scenarios [16]. Repetition and practice over time led to a reduction in the time taken to complete the moulage scenarios in certain studies [11, 16].

### 5.1 Experience and Task Execution

Experience and years in training seems to have a significant impact on the overall performance during a VR scenario. Colonna et al reported more decisions per minute were made by senior surgical residents compared to their junior counterparts during the VR simulation and the reported mortality was 92.3% for novices, falling to 25% for senior residents and 0% for consultant/attending surgeons [13]. This is similar to Pucher et al's study who reported more critical missteps in the novice groups and who took almost twice as long to manage the patients and had lower technical and nontechnical skills scores [20]. It also echoes Harrington et al who reported fewer fatal errors and incorrect decisions (statistically significant) in the instructor VR group when compared to the candidates [29]. Understandably, experience and exposure to managing a particular skill or scenario correlates with overall task performance [30]. However, this is offset with familiarity and use of the technology involved. VR provides an alternative adjunct to fill a niche in areas where procedures and skills may be emerging, are less frequently available or the stakes are significantly higher. VR offers a safe and controlled environment for junior members of surgical and trauma teams to practice their skills and knowledge.

### 5.2 Correlation With Gaming

Brenner et al reported a statistically significant shorter task time during microcatheter exchange in the REBOA VR group amongst participants who played video games regularly [11]. Interestingly, previous experience of playing video games did not demonstrate a difference in the general acceptance of new technology but did report enhanced self‐reflection in their clinical ability amongst gamers [13]. The majority of participants in Youngblood's study were not gamers and the study found no statistically significant difference in the performance of participants between the simulation and virtual world group. This highlights the need for larger studies as well as external validity of the software and hardware used amongst different groups.

The development of increasingly complex virtual scenarios, similar to current video games, can be utilized to adjust the difficulty of the task at hand. Couperus et al incorporated their physiology engine which factored in the ambient temperature and moisture in the surroundings to develop a complex and dynamic medical decision trainer capable of creating a multitude of patient based scenarios whilst maintaining the objective that is, to keep the patient alive for an established period of time [14]. Their group also created an autonomously running immersive VR capable of functioning without any instructor input and would be of benefit in military trauma practice where training opportunities could be limited. The concept is an adaptation of current gaming consoles which provide feedback in real‐time. In VR, the same concept can be used to add layers of complexity, alter the difficulty of scenarios and add further stressors to elicit the physiological stress response seen in the real world. In mass casualty incident management scenarios, avatars can be added as distractors under the guise of family members and news reporters to reflect the multi‐dimensional nature of managing trauma [20]. Furthermore, interaction with other members of the multidisciplinary team members in the VR setting is reflective of the real world, helping to improve overall teamwork and communication skills [16, 20, 21]. There are several studies comparing the technical skills in terms of laparoscopic skills, robotic surgery and endoscopy in gamers versus non‐gamers where findings have demonstrated improvement in some metrics including overall performance and a reduction in time to completion of tasks in gamers [31]. This has yet to be explored further in virtual reality‐based projects including correlation to task completion and personality types which warrant further studies.

### 5.3 Limitations

VR has its limitations as well. The studies included use a range of participants with significant differences in previous experience, access to training and familiarity with the technology. Use of convenience sampling and small sample sizes indicate that the reported results must be interpreted and accepted with caveats. Furthermore, each study has created and used their own in‐house programs which have not been re‐tested or validated in other environments and with other student/participant groups.

VR can offer audio‐visual cues however; tactile and haptic feedback integration still have a long way to go in developing its role in medical education. Arif et al. found no statistically significant difference in the tourniquet placement but did find the VR group had a higher number of participants who had failed as a result of improper tourniquet tightening when compared to their control counterparts even though both groups had the same baseline in‐person training before randomization [10]. This is similar to Proctor et al's reported increase in the cognitive load during the physical execution of tasks such as cannula insertion or cricothyroidotomy in the VR setting when compared to simulation and is in part due to the lack of haptic/tactile feedback which can be present in high fidelity mannequin‐based simulation. VR can visually and audibly create the stressful environments for challenging situations encountered in trauma but cannot provide the haptic/sensory feedback which is essential in successfully managing it and has yet to be developed further [10, 17]. Wearable haptics with cutaneous feedback encompassing proprioception, pressure, vibration, kinesthetic stimuli and tangential motion can improve the overall immersive experience but is still in its relative infancy and current VR headsets do not offer these without exponentially increasing the costs [32]. Future iterations of the hardware may incorporate better haptic feedback to allow expansion of the use of VR in developing and assessing technical skills in future. Whilst VR offers higher engagement and stimulation, it did not demonstrate short or long term retention of information when compared to traditional didactic teaching and further studies are needed to assess the long‐term impact of VR as a teaching modality [15]. VR can be associated with visuospatial motion sickness in the initial phases of use and the headsets are not always suitable for individuals who may wear glasses. This is likely to improve in future with the use of ultra‐thin optical elements, micro‐light emitting diodes and nanoscale engineered meta surfaces which could allow a better user experience [33]. Whilst the initial costs of acquiring the hardware are generally reasonable, the development of high‐fidelity software which could be validated and utilized on a large scale for training and assessment would not be insignificant. However, once developed–it could be easily integrated without significant barriers in mid to low‐income regions and further cost analyses would be of benefit. VR can be independent of geographical and resource constraints (i.e., staff, location, infrastructure, etc) and could offer an effective additional teaching modality to pre‐established methods in the rapidly evolving world of technological advancements.

The studies included in this review are isolated, pilot studies without future developments or follow‐up projects. This heterogeneity of software, hardware, methodology and metrics assessed makes it difficult to draw definitive conclusions and is echoed in other emerging literature pertaining to technologies when applied to trauma based teaching [7]. The complex interplay of cognitive, social and task‐oriented demands in trauma management require familiarization with its management. Repetition and training in trauma improves leadership, communication, emotional intelligence, teamwork and ultimately, patient outcomes [34, 35, 36]. Accessibility through novel evolving modalities could improve this further.

### 5.4 Future Scope of Use

As discussed, simulation, virtual reality and augmented reality have their benefits and limitations. Their use continues to be explored, particularly in developing decision making capabilities and improving skills. At present, its use is being explored in trauma management in areas of conflict, improving training in the management of pediatric trauma and expanding the use of these adjuncts to low/middle‐income countries (LMICs).

AR and VR can provide a useful adjunct for uptraining of paramedic reserves to improve pre‐hospital care and patient outcomes in areas of ongoing conflict where time and resources need to be rationalized but are offset by the need for healthcare professionals with the relevant skills [37]. This is also reflected in the management of pediatric trauma where the training required is expensive and somewhat limited, particularly in low and middle income countries [38]. Botelho et al tested a VR program replicating blunt head trauma and truncal injuries in a pediatric population with positive feedback [38]. A recent review evaluating the feasibility of medical education technologies in teaching trauma found significant disparities in being able to deliver training across high and LMIC's [39]. High fidelity simulation training kit is often expensive to buy and maintain. Its use requires training of staff, maintenance costs and has limited reusability. Interestingly, the review found virtual simulation and digital courses were generally more feasible, easy to integrate and were user‐friendly in LMIC's [39]. The general availability and improving affordability makes VR and AR a useful option which may form a greater proportion of training curricula in the future.

## 6 Conclusion

The use of VR in trauma‐based teaching and assessment is still in its early phase and not a replacement for simulation but it is a useful adjunct to established teaching methods. Current evidence surrounding virtual reality as an effective teaching modality is inconclusive and inconsistent. However, it is well perceived, feasible and users to date report very positive subjective feedback. Further robust studies with larger sample sizes and uniformity of the software and training levels will aid validity and could guide its use in the future.

## Author Contributions

**Manal Ahmad, Joseph Shalhoub, Alun Huw Davies:** project design, conceptualization. **Manal Ahmad, Mi‐Tra Tran, Basma Ahmad, Konstantinos Kavallieros:** data collection, analysis. **Manal Ahmad, Mi‐Tra Tran:** drafted the manuscript. **Joseph Shalhoub, Alun Huw Davies:** senior critical review of drafts. All authors approved the final version of this review.

## Conflicts of Interest

The authors declare no conflicts of interest.

## Data Availability

The data that support the findings of this study are available from the corresponding author upon reasonable request.

## Appendix A

### A.1

**Table A1:** Newcastle Ottawa scale for risk of bias.

Author	Newcastle‐Ottawa Scale
Arif et al.	Good
Bowyer et al.	Poor
Brenner et al.	Poor
Cohen et al.	Poor
Colonna et al.	Poor
Couperus et al.	Poor
Courteille et al.	Fair
Hainsworth et al.	Poor
Harrington et al.	Fair
Kiyozumi et al.	Poor
Proctor et al.	Good
Pucher et al.	Good
Youngblood et al.	Fair
